# Transversality of holomorphic maps into hyperquadrics

**DOI:** 10.1007/s00208-025-03134-5

**Published:** 2025-03-26

**Authors:** Xiaojun Huang, Weixia Zhu

**Affiliations:** 1 Department of Mathematics, Rutgers University, New Brunswick, NJ 08903 USA; 2 Faculty of Mathematics, University of Vienna, 1090 Vienna, Austria

## Abstract

We study holomorphic maps *F* from a smooth Levi non-degenerate real hypersurface $$ M_{\ell }\subset {\mathbb {C}}^n $$ into a hyperquadric $$ {\mathbb {H}}_{\ell '}^N $$ with signatures $$ \ell \le (n-1)/2 $$ and $$ \ell '\le (N-1)/2,$$ respectively. Assuming that $$ N - n < n - 1,$$ we prove that if $$ \ell = \ell ',$$ then *F* is either CR transversal to $$ {\mathbb {H}}_{\ell }^N $$ at every point of $$ M_{\ell },$$ or it maps a neighborhood of $$ M_{\ell } $$ in $$ {\mathbb {C}}^n $$ into $$ {\mathbb {H}}_{\ell }^N.$$ Furthermore, in the case where $$ \ell ' > \ell ,$$ we show that if *F* is not CR transversal at $$0\in M_\ell ,$$ then it must be transversally flat. The latter is best possible.

## Introduction

Let $M_1$ and $M_2$ be connected smooth CR hypersurfaces in ${{\mathbb {C}}}^n$ and ${{\mathbb {C}}}^N,$ respectively, with $2 < n \le N.$ Suppose that *F* is a holomorphic map from some small neighborhood $U \subset {{\mathbb {C}}}^n$ of $M_1$ into ${{\mathbb {C}}}^N$ such that $F(M_1) \subset M_2.$ We say that *F* is *totally degenerate* if there exists an open subset $V\subset U$ such that $F(V) \subset M_2.$ For $p\in M_1,$ we say *F* is *CR transversal* to $M_2$ at *p* if

$$\begin{aligned} T^{(1,0)}_{F(p)}M_2+dF\big (T^{(1,0)}_p{\mathbb {C}}^{n}\big )=T^{(1,0)}_{F(p)}{\mathbb {C}}^{N}. \end{aligned}$$

In a normalized coordinates, as we will demonstrate later, the CR transversality is equivalent to the non-vanishing property of the normal derivative of the normal component of the map.

Whether a holomorphic map is CR transversal is crucial in understanding the rigidity and extension problems of holomorphic maps. When both the target and the source manifolds are strongly pseudoconvex, CR transversality can be derived by applying the classical Hopf lemma (see a paper by Fornaess in [10]). When the target is not pseudoconvex, it becomes a difficult problem in general and has been studied extensively by many authors. For works in the equal dimensional case, see Baouendi–Rothschild [4], Huang [11, 12], Huang–Pan [13], Ebenfelt–Rothschild [7], Lamel–Mir [16], Ebenfelt–Son [8], and many references therein.

The study of the higher codimensional case, namely the case where $N > n,$ began with the work of Baouendi–Huang in [3], where it was proved that CR transversality always holds when the manifolds are hyperquadrics of the same signature for not-totally degenerate holomorphic maps. Baouendi–Ebenfelt–Rothschild [2] proved, under a rather general setting, that CR transversality holds in an open dense subset. See also the two papers by Huang–Zhang [14, 15] and a paper by Ebenfelt–Son [9].

While there exist examples [1] where CR transversality fails in a general setting, motivated by the transversality obtained in [3], Baouendi and the first author made the following conjecture:

### Conjecture

(Baouendi–Huang, 2005) Let $M_1 \subset {\mathbb {C}}^{n}$ and $M_2 \subset {\mathbb {C}}^{N}$ be two (connected) Levi non-degenerate real-analytic hypersurfaces with the same signature $0 < \ell \le (n-1)/2,$ where $2< n < N.$ Suppose *F* is a holomorphic map defined in a neighborhood *U* of $M_1,$ sending $M_1$ into $M_2.$ Then *F* is either a local CR embedding of $M_1$ into $M_2,$ or it is totally degenerate in the sense that it maps a neighborhood *U* of $M_1$ in ${\mathbb {C}}^{n}$ into $M_2.$

For the hypersurfaces $M_1$ and $M_2$ given in the conjecture, the hypothesis that *F* is CR transversal at $p \in M_1$ is equivalent to *F* being a CR embedding from a neighborhood of *p* in $M_1$ into $M_2.$ The first progress on the above conjecture was made in [15], where it was verified for the case when $M_2={{\mathbb {H}}}_\ell ^N$ and $N-n<(n-1)/2.$ Here we recall that a hyperquadric ${{\mathbb {H}}}_\ell ^N$ of signature $\ell$ in ${\mathbb {C}}^N$ is defined by

$$\begin{aligned} {{\mathbb {H}}}_\ell ^N:= \big \{(z, w)\in {{\mathbb {C}}}^{N-1}\times {{\mathbb {C}}}: {{\,\textrm{Im}\,}}w =|z|^2_\ell \big \}, \end{aligned}$$

where for any *n*-tuples *a* and *b*,  $\langle a, {\bar{b}}\rangle _{\ell }: = -\sum \limits _{j=1}^\ell a_j{\bar{b}}_j+ \sum _{j=\ell +1}^{n} a_j{\bar{b}}_j$ and $|a|^2_\ell = \langle a, {\bar{a}}\rangle _{\ell }.$

A major tool in the work of Huang–Zhang [15] is the rigidity theorem of Ebenfelt–Huang–Zaitsev [6] of CR embedding into quadrics. However, this result is not applicable when $N - n \ge (n-1)/2.$ In this paper, we build on a quantitative version [15] of a fundamental lemma originally introduced by the first author in [12], while also developing new ideas to replace the Ebenfelt–Huang–Zaitsev rigidity theorem. In the case where $\ell = \ell ',$ we establish the following result:

### Theorem 1.1

Let $M_\ell \subset {{\mathbb {C}}}^n$ be a smooth,  Levi non-degenerate real hypersurface with signature $0<\ell \le (n-1)/2.$ Suppose that *F* is a holomorphic map from an open neighborhood *U* of $M_\ell$ in ${{\mathbb {C}}}^n$ into ${{\mathbb {C}}}^N$ such that $F(M_\ell )\subset {\mathbb {H}}^N_\ell ,$ $N-n< n-1.$ Then *F* is either CR transversal to ${\mathbb {H}}_\ell ^N$ or maps a neighborhood of $M_\ell$ in *U* into ${\mathbb {H}}_\ell ^N.$

In the case where $\ell < \ell ' \le (N-1)/2,$ [1] established that if $M_1 = {\mathbb {H}}_\ell ^n$ and $M_2 = {\mathbb {H}}_{\ell '}^N,$ and *F* is not CR transversal at 0,  then under the signature condition $\ell ' < 2\ell ,$ *F* must be *transversally flat*, meaning that its normal component vanishes identically. Inspired by the proof of Theorem 1.1, we establish the following result. Unlike [1], our theorem imposes no restrictions on $\ell$ and $\ell ',$ but instead requires the codimension condition $N - n < n - 1.$ Moreover, we only assume that the source manifold is Levi non-degenerate.

### Theorem 1.2

Let $$ M_\ell \subset {\mathbb {C}}^n $$ be a smooth,  Levi non-degenerate real hypersurface with signature $$ 0 < \ell \le (n-1)/2.$$ Suppose *F* is a holomorphic map from an open neighborhood *U* of $$ M_\ell $$ in $$ {\mathbb {C}}^n $$ into $$ {\mathbb {C}}^N,$$ such that $$ F(M_\ell ) \subset {\mathbb {H}}^N_{\ell '} $$ and $$ F(0) = 0,$$ where $$ \ell \le \ell '.$$ Assume that $$ N - n < n - 1.$$ If *F* is not CR transversal at 0,  then *F* must be transversally flat,  or equivalently,  $$F(U)\subset \tilde{Q}_0$$ where $$\tilde{Q}_0$$ is the Segre variety of $${\mathbb {H}}^N_{\ell '}$$ at 0.

For a hyperquadric ${{\mathbb {H}}}^N_{\ell '}$ defined by $\tilde{\rho }\big ( (\tilde{z},\tilde{w}),\overline{(\tilde{z},\tilde{w})}):= {{\,\textrm{Im}\,}}\tilde{w} + \sum _{j=1}^{\ell '} |\tilde{z}_j|^2 - \sum _{j=\ell '+1}^{N-1} |\tilde{z}_j|^2 = 0$ in ${{\mathbb {C}}}^{N}$ with coordinates $(\tilde{z},\tilde{w})\in {{\mathbb {C}}}^{N-1}\times {{\mathbb {C}}},$ its Segre variety $\tilde{Q}_0$ at 0 is the complex analytic variety defined by $\tilde{\rho }((\tilde{z},\tilde{w}),0)=0$ or $\tilde{w}=0.$ The following two examples were first constructed by Baouendi–Ebenfelt–Huang in [1]. The first example shows that in Theorem 1.1, one does not have the CR transversality for not-totally degenerate holomorphic maps when $\ell '>\ell .$ The second example demonstrates that the result in Theorem 1.2 is sharp as one does not have transversal flatness when $N-n\ge n-1.$

### Example 1.3

Let $M_1 \subset {\mathbb {C}}^n$ be the hypersurface defined by $\rho = {{\,\textrm{Im}\,}}w + \sum _{j=1}^{\ell } |z_j|^2 - \sum _{j=\ell +1}^{n-1} |z_j|^2 = 0,$ and $M_2 \subset {\mathbb {C}}^{n+2}$ be the Levi non-degenerate hyperquadric defined by $\tilde{\rho }={{\,\textrm{Im}\,}}\tilde{w}+\sum _{j=1}^{\ell } |\tilde{z}_j|^2 - \sum _{j=\ell +1}^{n} |\tilde{z}_j|^2 + |\tilde{z}_{n+1}|^2.$ Consider the map $F: {\mathbb {C}}^n \rightarrow {\mathbb {C}}^{n+2}$ given by:

$$\begin{aligned} F(z,w) = \left( 2z_1^2, 2z_1z_2, \ldots , 2z_1z_{n-1}, z_1(1+iw), z_1(1-iw), 0\right) . \end{aligned}$$

A direct computation shows that $\tilde{\rho }\circ F= 4|z_1|^2 \rho ,$ which implies that CR transversality fails at $z_1 = 0$ though $F({{\mathbb {C}}}^n)\not \subset M_2.$ In this example, the signature difference is $\ell ' - \ell = 1,$ and the codimension is $N - n = 2.$ This demonstrates that the assumption $\ell ' = \ell$ in Theorem 1.1 cannot be dropped.

### Example 1.4

Let $M_1 \subset {\mathbb {C}}^3: \rho ={{\,\textrm{Im}\,}}w+|z_1|^2-|z_2|^2,$ and $M_2 \subset {\mathbb {C}}^{5}: \tilde{\rho }={{\,\textrm{Im}\,}}\tilde{w}+|\tilde{z}_1|^2+|\tilde{z}_2|^2-|\tilde{z}_3|^2-|\tilde{z}_4|^2.$ Consider the map $F: {\mathbb {C}}^3 \rightarrow {\mathbb {C}}^{5}$ given by:

$$\begin{aligned} F(z,w)=\left( z_1+\frac{z_1^2}{2}-\frac{i}{4}w,z_2- \frac{z_1z_2}{2},z_1-\frac{z_1^2}{2}+\frac{i}{4}w,z_2+ \frac{z_1z_2}{2},z_1w\right) . \end{aligned}$$

We have $\tilde{\rho }\circ F = (z_1 + \bar{z}_1) \rho ,$ indicating that CR transversality fails when ${{\,\textrm{Re}\,}}z_1 = 0.$ However, *F* is not transversally flat. Since the codimension in this example satisfies $N - n = n - 1,$ this demonstrates the sharpness of the condition $N - n < n - 1$ in Theorem 1.2.

Other main tools employed in this papers include recent study of convergence of formal maps into real algebraic subsets in Meylan–Mir–Zaistev [20] and more recent work in Lamel-Mir [17].

## Proof of main theorems

We first assume that $M_\ell \subset {\mathbb {C}}^n$ is a real analytic hypersurface with signature $\ell ,$ where $0 < \ell \le (n-1)/2.$ Let $p \in M_\ell .$ After a holomorphic translation, assume $p = 0 \in M_\ell .$ Let $V \subset U$ be a small neighborhood of $0 \in M_\ell$ in ${\mathbb {C}}^n.$ Following a holomorphic change of coordinates as described in [5], near the origin, $M_\ell$ and ${\mathbb {H}}_{\ell '}^N$ can be locally represented by

$$\begin{aligned} M_\ell =\left\{ (z,w)\in {{\mathbb {C}}}^{n-1}\times {{\mathbb {C}}}: {{\,\textrm{Im}\,}}w=-\sum _{j=1}^\ell |z_j|^2+\sum _{j=\ell +1}^{n-1}|z_j|^2+R(z,{\bar{z}}, {{\,\textrm{Re}\,}}w) \right\} \end{aligned}$$

and

$$\begin{aligned} {\mathbb {H}}_{\ell '}^N=\left\{ (\tilde{z},\tilde{w})\in {{\mathbb {C}}}^{N-1}\times {{\mathbb {C}}}: {{\,\textrm{Im}\,}}\tilde{w}=-\sum _{j=1}^{\ell '}|\tilde{z}_j|^2+\sum _{j=\ell '+1}^{N-1}|\tilde{z}_j|^2\right\} . \end{aligned}$$

Here, *R* is formulated as a power series with respect to *z* and ${\bar{z}}$ with coefficients being power series in ${{\,\textrm{Re}\,}}w.$ Specifically, it can be written as

2.1 $$\begin{aligned} R(z,{\bar{z}}, {{\,\textrm{Re}\,}}w)=\sum _{|\alpha |,|\beta |\ge 2}R_{\alpha ,\beta }(z,{\bar{z}}, {{\,\textrm{Re}\,}}w), \end{aligned}$$

with the property that $R_{\alpha ,\beta }(tz,\tau {\bar{z}}, {{\,\textrm{Re}\,}}w)=t^{\alpha }\tau ^{\beta }R_{\alpha ,\beta }(z,{\bar{z}}, {{\,\textrm{Re}\,}}w)$ for all $t,\tau \in {{\mathbb {C}}}.$ For $\ell <\ell ',$ we define

$$\begin{aligned} {\mathbb {H}}_{\ell ,\ell ',n}^N:=\left\{ (\tilde{z},\tilde{w})\in {{\mathbb {C}}}^{N}: {{\,\textrm{Im}\,}}\tilde{w}=-\sum _{j=1}^\ell |\tilde{z}_j|^2+\sum _{j=\ell +1}^{n-1}|\tilde{z}_j|^2-\sum _{j=n}^{n+\ell '-\ell -1}|\tilde{z}_j|^2+\sum _{j=n+\ell '-\ell }^{N-1}|\tilde{z}_j|^2 \right\} . \end{aligned}$$

Notice that ${\mathbb {H}}_{\ell ,\ell ',n}^N$ is holomorphically equivalent to ${\mathbb {H}}_{\ell '}^N$ by a linear map. From this point on, without causing confusion, ${\mathbb {H}}_{\ell '}^N$ in the following text will actually refer to ${\mathbb {H}}_{\ell ,\ell ',n}^N.$

Adhering to the notation in [5], we assign the weight of *z* to be 1 and the weight of ${{\,\textrm{Re}\,}}w$ to be 2. For $m\in {{\mathbb {N}}},$ a function $h(z,{\bar{z}}, {{\,\textrm{Re}\,}}w)$ defined in *V* is said to be $o_{\text {wt}}(m)$ if $h(tz, t{\bar{z}},t^2 {{\,\textrm{Re}\,}}w)/|t|^m\rightarrow 0$ uniformly as $t\rightarrow 0.$ Additionally, we denote by $h^{(m)}(z, {\bar{z}},{{\,\textrm{Re}\,}}w)$ a weighted homogeneous polynomial of weighted degree *m*. Functions following certain weighted homogeneity conditions are denoted with specific notations. For instance, when $h^{(m)}(z, {\bar{z}},{{\,\textrm{Re}\,}}w)$ is a weighted holomorphic polynomial of weighted degree *m*,  we also write it as $h^{(m)}(z, w)$ with *z* having a weighted degree 1 and *w* having a weighted degree 2.

Let *F* be a holomorphic map defined in *U* sending $(M_\ell ,0)$ into $({\mathbb {H}}_{\ell '}^N,0).$ Write

$$\begin{aligned} F:=({\widetilde{f}},g)=(f,\phi ,g)=(f_1,\ldots ,f_{n-1}, \phi _1,\ldots ,\phi _{N-n},g). \end{aligned}$$

Suppose that *F* is CR transversal at 0,  meaning $\frac{\partial g}{\partial w}(0) = \sigma \lambda ^2 \ne 0,$ where $\lambda > 0$ and $\sigma = \pm 1.$ When $\ell < (n-1)/2,$ we can always set $\sigma = 1.$ However, when $\ell = (n-1)/2,$ it is possible to have $\sigma = -1.$ In this case, replacing *F* with $F \circ \tau _{\frac{n-1}{2}}$ allows us to take $\sigma = 1,$ where $\tau _{\frac{n-1}{2}}$ is a coordinate transformation defined by $\tau _{\frac{n-1}{2}}\left( z_1,\ldots ,z_{\frac{n-1}{2}}, z_{\frac{n-1}{2}+1},\ldots ,z_{n-1},w\right) =\left( z_{\frac{n-1}{2}+1},\ldots ,\right.$ $\left. z_{n-1}, z_1,\ldots ,z_{\frac{n-1}{2}},-w\right) .$ Therefore, we may assume $\ell \le (n-1)/2$ and $\sigma = 1$ throughout.

Applying a normalization from [3, §2], we express *F* as:

2.2 $$\begin{aligned} \begin{aligned}&{\widetilde{f}}(z,w)= zA+\textbf{a}w+O(|(z,w)|^2)=\lambda zA_\lambda +\textbf{a}w+O(|(z,w)|^2),\\&g(z,w)=\lambda ^2w+O(|(z,w)|^2), \end{aligned}\qquad \end{aligned}$$

where $\textbf{a}$ is an $(N-1)$-row vector, and $A=\lambda A_\lambda$ is an $(n-1)\times (N-1)$ matrix of rank $n-1,$ satisfying that

2.3 $$\begin{aligned} A E_{(\ell ,\ell ',n-1,N-1)}{\overline{A}}^{\,t}=\lambda ^2 E_{(\ell ,\ell ,n-1,n-1)}. \end{aligned}$$

In this context, $E_{(\ell ,\ell ',m,n)}$ denotes an $n \times n$ diagonal matrix with the first $\ell$ diagonal elements as well as elements at position *m* to $m+\ell '-\ell -1$ equal to $-1$ and the remaining diagonal elements equal to 1. More precisely,

$$\begin{aligned} E_{(\ell ,\ell ',n-1,N-1)} = \begin{pmatrix} -I_{\ell } & \quad 0 & \quad 0 & \quad 0 \\ 0 & \quad I_{n-\ell -1} & \quad 0 & \quad 0 \\ 0 & \quad 0 & \quad -I_{\ell '-\ell } & \quad 0 \\ 0 & \quad 0 & \quad 0 & \quad I_{N-n-\ell '+\ell } \end{pmatrix} \end{aligned}$$

Write

$$\begin{aligned} A_\lambda =\begin{pmatrix} \alpha _1\\ \vdots \\ \alpha _{n-1} \end{pmatrix} \end{aligned}$$

where $\alpha _i:=(a_{i1},\ldots ,a_{i(N-1)}),$ $a_{ij}\in {\mathbb {C}},$ $i=1,\ldots , n-1.$ From (2.3), we have

2.4 $$\begin{aligned} A_{{\lambda }} E_{(\ell ,\ell ',n-1,N-1)} \overline{A}_{{\lambda }}^{\,t}=E_{(\ell ,\ell ,n-1,n-1)}. \end{aligned}$$

Let $E:=E_{(\ell ,\ell ',n-1,N-1)}.$ For two $(N-1)$-dimensional complex vectors *X* and *Y*,  we let

$$\begin{aligned} \langle X,\overline{Y}\rangle _E:=XE_{(\ell ,\ell ',n-1,N-1)} \overline{Y}^{\,t},\qquad |X|_E^2:=\langle X,\overline{X}\rangle _E. \end{aligned}$$

It follows from (2.4) that $|\alpha _j|_E^2=-1$ when $1\le j\le \ell ,$ $|\alpha _j|_E^2=1$ when $\ell +1\le j\le n-1,$ and $\langle \alpha _j,\overline{\alpha }_k\rangle _E=0$ when $j\ne k.$ We refer to $\alpha _j$ as a “negative” vector if $|\alpha _j|_E^2 < 0,$ and as a “positive” vector if $|\alpha _j|_E^2 > 0.$ The following theorem shows that $A_\lambda$ can be extended to an invertible $(N-1)\times (N-1)$ matrix ${\widetilde{A}}_{\lambda }$ with the maximum absolute values of its elements bounded by a constant depending only on the norm of $A_\lambda$:

### Theorem 2.1

Let $\alpha _1,\ldots ,\alpha _{n-1}$ be $(N-1)$-dimensional row complex vectors of $A_{\lambda }$ that satisfy (2.4). Then there exists an $(N-1)\times (N-1)$ matrix

$$\begin{aligned} {\widetilde{A}}_{\lambda } = \begin{pmatrix} A_{\lambda }\\ \alpha _{n} \\ \vdots \\ \alpha _{N-1} \end{pmatrix}, \end{aligned}$$

such that

2.5 $$\begin{aligned} {\widetilde{A}}_\lambda E_{(\ell ,\ell ',n-1,N-1)} \overline{{\widetilde{A}}}_{\lambda }^{\,t}=E_{(\ell ,\ell ',n-1,N-1)}. \end{aligned}$$

Furthermore,  if we let $$M:= {\text {max}}_{\begin{array}{c} \scriptscriptstyle 1\le i\le n-1\\ \scriptscriptstyle 1\le j\le N-1 \end{array}}\left\{ |a_{ij}|,1\right\} ,$$ then

2.6 $$\begin{aligned} |a_{ij}|\le \sqrt{1+\ell '}\,2^{n(\ell '-\ell )}M, \end{aligned}$$

for all $i > n-1$ and $1\le j\le N-1.$

### Proof

The existence of $\widetilde{A}_{\lambda }$ satisfying the estimate (2.5) is known (see [3]). It remains to show that any $\widetilde{A}_{\lambda }$ satisfying (2.5) also satisfies (2.6). We split the proof into two cases:

**Case 1**: $\ell = \ell '.$ In this case, all vectors whose lengths need to be shown to be controllable are positive. By multiplying both sides of (2.5) on the left by $\overline{{\widetilde{A}}}_{\lambda }^{\,t}E_{(\ell ,\ell ,n-1,N-1)},$ we get

$$\begin{aligned} \overline{{\widetilde{A}}}_{\lambda }^{\,t}E_{(\ell ,\ell ,n-1,N-1)}{\widetilde{A}}_\lambda E_{(\ell ,\ell ,n-1,N-1)}\overline{{\widetilde{A}}}_{\lambda }^{\,t} = \overline{{\widetilde{A}}}_{\lambda }^{\,t}E_{(\ell ,\ell ,n-1,N-1)}E_{(\ell ,\ell ,n-1,N-1)} = \overline{{\widetilde{A}}}_{\lambda }^{\,t}. \end{aligned}$$

Since $\overline{{\widetilde{A}}}_{\lambda }^{\,t}$ is invertible, multiplying both sides on the right by $\big (\overline{{\widetilde{A}}}_{\lambda }^{\,t}\big )^{-1}E_{(\ell ,\ell ,n-1,N-1)}$ yields

2.7 $$\begin{aligned} \overline{{\widetilde{A}}}_{\lambda }^{\,t}E_{(\ell ,\ell ,n-1,N-1)}{\widetilde{A}}_{\lambda } = E_{(\ell ,\ell ,n-1,N-1)}, \end{aligned}$$

which implies

$$\begin{aligned} -\sum _{i=1}^{\ell }|a_{ij}|^2 + \sum _{i=\ell +1}^{N-1}|a_{ij}|^2 = \delta _j^\ell \quad \text {for all } 1 \le j \le N-1, \end{aligned}$$

where $\delta _j^\ell =-1$ when $j\le \ell ,$ $\delta _j^\ell =1$ when $j> \ell .$ Thus, for $i > \ell ,$ we obtain

$$\begin{aligned} |a_{ij}|\le \sqrt{\delta _j^\ell + \sum _{i=1}^{\ell }|a_{ij}|^2 }\le \sqrt{1 + \ell M^2}\le \sqrt{1+\ell }M. \end{aligned}$$

This completes the proof for the case $\ell = \ell '.$

**Case 2**: $\ell < \ell '.$ In this case, if we are able to construct all the necessary negative unit vectors $\alpha _{n},\alpha _{n+1},\ldots ,\alpha _{n+\ell '-\ell -1},$ the situation reduces to Case 1. Therefore, our goal is to construct these negative vectors. Let us begin by constructing $\alpha _{n}$:

Define an *N*-dimensional row vector

$$\begin{aligned} \beta _{n} = (b_{n1}, \ldots , b_{n\ell }, 0_{\ell +1}, \ldots , 0_{n-1}, b_{nn}, \ldots , b_{n(n+\ell ' - \ell -1)}, 0_{n+\ell ' - \ell }, \ldots , 0_{N-1}), \end{aligned}$$

such that $|\beta _{n}|_{E}^2 = -1.$ This gives

$$\begin{aligned} \sum _{j=1}^{\ell } |b_{nj}|^2 + \sum _{j=1}^{\ell ' - \ell } |b_{n(n-1+j)}|^2 = 1. \end{aligned}$$

Since $\ell < \ell ',$ it is clear that we can choose $\beta _{n}$ such that it is orthogonal to all $\alpha _j$ for $1 \le j \le \ell ,$ i.e., $\langle \beta _{n}, \alpha _j \rangle _E = 0.$ Now, define

$$\begin{aligned} \beta ^{(1)}_{n} = \beta _{n} - \langle \beta _{n}, \overline{\alpha }_{\ell +1} \rangle _E \, \alpha _{\ell +1}. \end{aligned}$$

It is easy to verify that $\langle \beta ^{(1)}_{n}, \alpha _j \rangle _E = 0$ for all $1 \le j \le \ell +1.$ Moreover, we have

$$\begin{aligned} -1 = |\beta _{n}|_{E}^2 = |\beta ^{(1)}_{n}|_{E}^2 + \big |\langle \beta _{n}, \overline{\alpha }_{\ell +1} \rangle _E\big |^2, \end{aligned}$$

which implies

$$\begin{aligned} |\beta ^{(1)}_{n}|_{E}^2 = -1 - \big |\langle \beta _{n}, \overline{\alpha }_{\ell +1} \rangle _E\big |^2. \end{aligned}$$

Let

$$\begin{aligned} \alpha ^{(1)}_{n}: = \frac{\beta ^{(1)}_{n}}{\sqrt{1 + \big |\langle \beta _{n}, \overline{\alpha }_{\ell +1} \rangle _E\big |^2}}. \end{aligned}$$

Thus, $|\alpha ^{(1)}_{n}|_{E}^2 = -1,$ and it satisfies the orthogonality conditions $\langle \alpha ^{(1)}_{n}, \alpha _j \rangle _E = 0$ for all $1 \le j \le \ell +1.$ Additionally, we have the following bound:

$$\begin{aligned} |a^{(1)}_{nj}| = \frac{\left| b_{nj} - \langle \beta _{n}, \overline{\alpha }_{\ell +1} \rangle _E \, a_{(\ell +1)j}\right| }{\sqrt{1 + \big |\langle \beta _{n}, \overline{\alpha }_{\ell +1} \rangle _E\big |^2}} \le \frac{1 + |\langle \beta _{n}, \overline{\alpha }_{\ell +1} \rangle _E| \, M}{\sqrt{1 + \big |\langle \beta _{n}, \overline{\alpha }_{\ell +1} \rangle _E \big |^2}} \le 1 + M\le 2M, \end{aligned}$$

for all $1 \le j \le N-1.$ We then proceed similarly for each $1 \le k \le n - \ell - 2.$ Let

$$\begin{aligned} \beta ^{(k+1)}_{n} = \alpha ^{(k)}_{n} - \langle \alpha ^{(k)}_{n}, \overline{\alpha }_{\ell +1+k} \rangle _E \, \alpha _{\ell +1+k}, \end{aligned}$$

and

$$\begin{aligned} \alpha ^{(k+1)}_{n}:= \frac{\beta ^{(k+1)}_{n}}{\sqrt{1 + \big |\langle \alpha ^{(k)}_{n}, \overline{\alpha }_{\ell +1+k} \rangle _E\big |^2}}. \end{aligned}$$

It is easy to check that $|\alpha ^{(k+1)}_{n}|_E^2 = -1,$ and $\langle \alpha ^{(k+1)}_{n}, \overline{\alpha }_j \rangle _E = 0$ for all $1 \le j \le \ell +k+1.$ Furthermore, for all $1 \le j \le N-1$:

$$\begin{aligned} |a^{(k+1)}_{nj}| \le 2^{k+1} M. \end{aligned}$$

Setting $$\alpha _{n}:= \alpha ^{(n-\ell -1)}_{n},$$ then $$|a_{nj}|\le 2^{n-\ell -1}M< 2^n M,$$ $$1\le j\le N-1.$$

The construction of the remaining negative unit vectors $\alpha _{n+1}, \ldots , \alpha _{n+\ell ' - \ell -1}$ follows the same process. Moreover, for each $0\le h\le \ell '-\ell -1,$ we have $|a_{(n+h)j}|\le 2^{n(\ell '-\ell )}M$ for all $1\le j\le N-1.$ The proof is complete by what we did in Case 1. $\square$

We now let ${\widetilde{A}}_\lambda$ be the unitary matrix obtained by extending $A_\lambda$ according to the theorem above satisfying (2.7). We can rewrite *F* as:

2.8 $$\begin{aligned} \begin{aligned}&{\widetilde{f}}(z,w)= \lambda (z,0_{\phi }){\widetilde{A}}_\lambda +\textbf{a}w+O(|(z,w)|^2),\\&g(z,w)=\lambda ^2w+O(|(z,w)|^2), \end{aligned} \end{aligned}$$

where $0_{\phi }$ is the zero vector in ${{\mathbb {C}}}^{N-n}.$ Let $s^*_{\lambda ,{\widetilde{A}}_\lambda }$ be an invertible scaling map of ${{\mathbb {C}}}^N$ given by

2.9 $$\begin{aligned} s^*_{\lambda ,{\widetilde{A}}_\lambda }({\tilde{z}},\tilde{w})=\left( \frac{1}{\lambda }{\tilde{z}} {\widetilde{A}}_\lambda ^{-1},\frac{1}{\lambda ^2}{\tilde{w}}\right) . \end{aligned}$$

Define

$$\begin{aligned} F^*= ({\widetilde{f}}^*, g^*) =(f^*,\phi ^*, g^*):= s^*_{\lambda ,{\widetilde{A}}_\lambda } \circ F \end{aligned}$$

and

$$\begin{aligned} \textbf{a}^* = (\textbf{a}^*_f, \textbf{a}^*_\phi ):= s^*_{\lambda ,{\widetilde{A}}_\lambda } \circ \textbf{a}, \end{aligned}$$

from which $F^*$ has the following expressions:

$$\begin{aligned} \begin{aligned}&f^*(z,w)= z+\textbf{a}^*_fw+O(|(z,w)|^2),\\&\phi ^*(z,w)=\textbf{a}^*_\phi w+O(|(z,w)|^2),\\&g^*(z,w)=w+O(|(z,w)|^2). \end{aligned} \end{aligned}$$

Let $r^*={{\,\textrm{Re}\,}}\dfrac{\partial ^2g^*}{\partial w^2}(0)$ and let $G_{r^*,\textbf{a}^*}\in \hbox {Aut}_0({{\mathbb {H}}}^N_{\ell })$ given by

2.10 $$\begin{aligned} \begin{aligned} G_{r^*,\textbf{a}^*}(z^*,w^*)=\dfrac{(z^*-\textbf{a}^*w^*,\, w^*)}{1+2i\langle z^*,\overline{\textbf{a}^*}\rangle _E +(r^*-i|\textbf{a}^*|^2_E)w^*}. \end{aligned} \end{aligned}$$

Let

$$\begin{aligned} F^{\sharp }=({\widetilde{f}}^\sharp ,g^\sharp )=(f^\sharp ,\phi ^\sharp , g^\sharp ):=G_{r^*,\textbf{a}^*}\circ F^*=G_{r^*,\textbf{a}^*}\circ s^*_{\lambda ,{\widetilde{A}}_\lambda } \circ F. \end{aligned}$$

Then $F^\sharp$ can be written as

2.11 $$\begin{aligned} \begin{aligned}&f^\sharp (z,w)= z+\frac{1}{2}f^{\sharp (1,0)}(z)w+o_{\text {wt}}(3),\\&\phi ^\sharp (z,w)=\phi ^{\sharp (2,0)}(z)+o_{\text {wt}}(2),\\&g^\sharp (z,w)=w+o_{\text {wt}}(4), \end{aligned} \end{aligned}$$

with

2.12 $$\begin{aligned} \big \langle f^{\sharp (1,0)}(z), {\bar{z}}\big \rangle _E|z|^2_{\ell }=|\phi ^{\sharp (2,0)}(z)|_{\ell '-\ell }^2-R_{2,2}(z,{\bar{z}}, 0), \end{aligned}$$

where $f^{\sharp (1,0)}(z)$ represents a $(n-1)$-dimensional vector-valued linear function in *z* without a constant term, and $\phi ^{\sharp (2,0)}(z)$ is a $(N-n)$-dimensional vector-valued function of homogeneous in *z* of degree two. (2.12) is often referred to as the Chern–Moser equation or Gauss equation, once we assume that $R_{2,2}$ satisfies the trace-free condition.

### Proof of Theorem 1.2

We start with the assumption that *F* is not CR transversal at 0,  which gives $\dfrac{\partial g}{\partial w}(0) = 0.$ If *F* is totally degenerate, then there exists a neighborhood *V* of 0 in ${{\mathbb {C}}}^n,$ such that

2.13 $$\begin{aligned} g(z,w)-\overline{g}(\chi ,\tau )=2i\langle \widetilde{f}(z,w),\overline{\widetilde{f}}(\chi ,\tau )\rangle _E \end{aligned}$$

holds for all $(z,w),(\chi ,\tau )\!\in \! V.$ Letting $(\chi ,\tau )\!=\!0$ in (2.13), we obtain that $g(z,w)\!\equiv \! 0.$

Now, assuming that *F* is not totally degenerate, according to [2, Theorem 1.1], for such an *F*,  the set of points where CR transversality holds forms an open dense subset in $M_\ell .$ We then choose a sequence of points $\{p_k\} \in M_\ell$ such that $p_k \rightarrow 0$ and *F* is CR transversal at each $p_k.$ As in [3], for each $p_k$ close to the origin, we can associate a map $F_{p_k}$ from a small neighborhood of 0 in $M_{p_k}$ to ${\mathbb {H}}_{\ell '}^N$ with $F_{p_k}(0)=0,$ and $F_{p_k}$ is CR transversal to ${\mathbb {H}}_{\ell '}^N$ at 0 for $p_k$ close to 0. Let’s explain it through the following diagrams: 
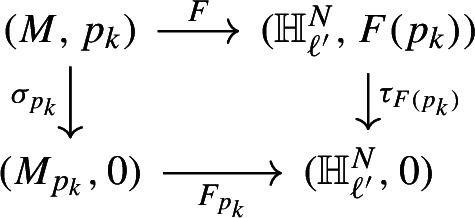
 Clearly, $F_{p_k}=(f_{p_k},\phi _{p_k}, g_{p_k})=\tau _{F(p_k)}\circ F\circ \sigma _{p_k}^{-1}.$ Here $\sigma _{p_k}$ is a biholomorphic maps such that $\sigma _{p_k}(p_k)=0,$ depend smoothly on $p_k.$ And $\tau _{F(p_k)}\in \text {Aut}({\mathbb {H}}_{\ell '}^N)$ is given by

$$\begin{aligned} \tau _{F(p_k)}({\tilde{z}},{\tilde{w}})=\Big ({\tilde{z}}-\widetilde{f}(p_k)), {\tilde{w}}-\overline{g(p_k)}-2i\langle {\tilde{z}}, \overline{\widetilde{f}(p_k)}\,\rangle _{E}\,\Big ). \end{aligned}$$

Furthermore, $M_{p_k}$ can be written as

$$\begin{aligned} M_{p_k}=\{(z,w)\in {{\mathbb {C}}}^{n-1}\times {{\mathbb {C}}}: {{\,\textrm{Im}\,}}w=|z|_\ell ^2+R_{p_k}(z,{\bar{z}}, {{\,\textrm{Re}\,}}w) \}, \end{aligned}$$

where $R_{p_k}$ is normalized to the 4-th order, having the same property as in (2.1).

For each $p_k,$ let $\dfrac{\partial g_{p_k}}{\partial w}(0)=\lambda _{k}^2.$ Notice that $\lambda _{k}\rightarrow 0$ since $g_w(0)=0.$ We now apply the normalization procedure outlined in (2.8)–(2.11) to $F_{p_k}.$ It follows that $F_{p_k}$ can be represented as:

$$\begin{aligned} \begin{aligned}&{\widetilde{f}}_{p_k}(z,w)= \lambda _{k}(z,0_{\phi })\widetilde{A}_{k}+{\textbf{a}_{k}}w+O(|(z,w)|^2),\\&g_{p_k}(z,w)=\lambda _{k}^2w+O(|(z,w)|^2). \end{aligned} \end{aligned}$$

Taking *k* to infinity, from Theorem 2.1, after passing to a subsequence if needed, we have the following convergence:

2.14 $$\begin{aligned} \begin{aligned}&\lambda _k\widetilde{A}_{k}\rightarrow \widetilde{A},&\quad \textbf{a}_{k}\rightarrow \textbf{a},&\quad {\widetilde{f}}_{p_k}\rightarrow {\widetilde{f}}, \quad&g_{p_k}\rightarrow g, \end{aligned} \end{aligned}$$

where $\widetilde{A}$ is a certain $(N-1)\times (N-1)$ matrix, $\textbf{a},$ ${\widetilde{f}},$ *g* are from the initial map (2.8), and hence uniformly bounded. It is noteworthy that $\widetilde{A}$ represents an $(N-1)\times (N-1)$ matrix, which is a bounded extension of *A* in (2.2). Let $F_{p_k}^*=s^*_{\lambda _k,A_k} \circ F_{p_k}$ and $F_{p_k}^{\sharp }=G_{r_k^*,\textbf{a}_k^*}\circ F_{p_k}^*,$ where $s^*_{\lambda _k,A_k}$ and $G_{r_k^*,\textbf{a}_k^*}$ are given by (2.9) and (2.10), respectively. Then

2.15 $$\begin{aligned} F_{p_k}^{\sharp }=(\widetilde{f}^{\sharp }_{p_k},g^{\sharp }_{p_k})&=\dfrac{({\widetilde{f}}_{p_k}^*-\textbf{a}_k^*g_{p_k}^*,\, g_{p_k}^*)}{1+2i\langle {\widetilde{f}}_{p_k}^*,\overline{\textbf{a}_k^*}\rangle _E +(r_k^*-i|\textbf{a}_k^*|^2_E)g_{p_k}^*}\nonumber \\&=\dfrac{\big (\lambda _k^{-1}({\widetilde{f}}_{p_k}-\lambda _k^{-2}\textbf{a}_kg_{p_k})\widetilde{A}_k^{-1},\, \lambda _k^{-2}g_{p_k}\big )}{1+\lambda _k^{-2}\cdot 2i\langle {\widetilde{f}}_{p_k},\overline{\textbf{a}}_k\rangle _E +\lambda _k^{-4}(r_k-i|\textbf{a}_k|^2_E)g_{p_k}}, \end{aligned}$$

where $\textbf{a}_k^*=s^*_{\lambda _k,A_k} \circ \textbf{a}_k,$ $r_k^*={{\,\textrm{Re}\,}}\dfrac{\partial ^2g_{p_k}^*}{\partial w^2}(0),$ and $r_k={{\,\textrm{Re}\,}}\dfrac{\partial ^2g_{p_k}}{\partial w^2}(0).$ Moreover, we obtain similar expressions for $F_{p_k}^{*}$ and $F_{p_k}^{\sharp }$ as in (2.8) and (2.11), respectively. In particular, we have

2.16 $$\begin{aligned} \begin{aligned}&f_{p_k}^\sharp (z,w)= z+\frac{i}{2}f_k^{\sharp (1,0)}(z)w+o_{\text {wt}}(3),\\&\phi _{p_k}^\sharp (z,w)=\phi _k^{\sharp (2,0)}(z)+o_{\text {wt}}(2),\\&g_{p_k}^\sharp (z,w)=w+o_{\text {wt}}(4), \end{aligned} \end{aligned}$$

with

2.17 $$\begin{aligned} \big \langle f_{k}^{\sharp (1,0)}(z), {\bar{z}}\big \rangle _E|z|^2_{\ell }=|\phi _{k}^{\sharp (2,0)}(z)|_{\ell '-\ell }^2-(R_{p_k})_{2,2}(z,{\bar{z}}, 0). \end{aligned}$$

Let $F^{\sharp (m)}_{p_k}$ denote the terms of weighted degree *m* in $F_{p_k}^{\sharp }.$ We claim that the $F_{p_k}^{\sharp }$ constructed above satisfies

2.18 $$\begin{aligned} \Vert g^{\sharp (m)}_{p_k}\Vert ,\Vert f^{\sharp (m)}_{p_k}\Vert , \Vert (|\phi ^\sharp _{p_k}|_{\ell '-\ell }^2)^{(m)}\Vert \le C_m \end{aligned}$$

for each $m \in {{\mathbb {N}}},$ provided that $N - n < n - 1.$ Write

$$\begin{aligned} \phi ^\sharp _{p_k}(z,w)=\sum _{\alpha ,\mu }(\phi ^\sharp _{p_k})_{\alpha ,\mu }(z)w^\mu \end{aligned}$$

where $(\phi ^\sharp _{p_k})_{\alpha ,\mu }$ is a homogeneous polynomial of degree $\alpha$ in *z*. Let

2.19 $$\begin{aligned} (A^{\sharp }_{p_k})_{\alpha \mu \beta \gamma }(z)=\sum _{j=1}^{N-n}\delta _j (\phi ^\sharp _{p_k,j})_{\alpha ,\mu }(z)\overline{(\phi ^\sharp _{p_k,j})_{\beta ,\gamma }(z)} \end{aligned}$$

with $\delta _j=-1$ for $j\le \ell '-\ell$ and $\delta _j=1$ for $j>\ell '-\ell .$ Then from (2.18) we actually have $\Vert (A^{\sharp }_{p_k})_{\alpha \mu \beta \gamma }\Vert \le C_m$ for $\alpha +\beta +2\mu +2\gamma \le m$. Here, $C_m$ is a constant independent of $p_k$ but may depends on *m*. In the case where $\ell ' = \ell ,$ it was shown in [15] that $|F^{\sharp (m)}_{p_k}| \le C_m$ by applying a quantitative fundamental result of [12] (see [15, Lemma 6]). Without assuming the same signature, we will demonstrate that the same idea works for the boundedness of $|g^{\sharp (m)}_{p_k}|$ and $|f^{\sharp (m)}_{p_k}|.$ This proof follows more or less the same method:

Note that, according to (2.16), for $m \le 4,$ we have the bounds $|g^{\sharp (m)}_{p_k}|, |f^{\sharp (m-2)}_{p_k}| \le 1$ and $|(|\phi ^\sharp _{p_k}|_{\ell '-\ell }^2)^{(m-1)}| = 0.$ Additionally, by applying a quantitative fundamental result of [12] (see [15, Lemma 6]) to (2.17), we obtain that $\Vert f^{\sharp (3)}_{p_k}\Vert , \Vert (|\phi ^\sharp _{p_k}|_{\ell '-\ell }^2)^{(4)}\Vert \le C$ under the condition $N-n<n-1.$ We will then prove this claim by induction, focusing on the boundedness of $\Vert g^{\sharp (m)}_{p_k}\Vert ,$ $\Vert f^{\sharp (m-1)}_{p_k}\Vert$ and $\Vert (A^{\sharp }_{p_k})_{\alpha \mu \beta \gamma }\Vert$ for $\alpha +\beta +2\mu +2\gamma \le m,$ where $(A^{\sharp }_{p_k})_{\alpha \mu \beta \gamma }$ is defined in (2.19). It follows from $F^\sharp _{p_k}(M_{p_k})\subset {\mathbb {H}}_{\ell '}^N$ that

2.20 $$\begin{aligned}  &   {{\,\textrm{Im}\,}}g^\sharp _{p_k}(z, u + i(|z|_\ell ^2 + o_{\text {wt}}(3))) - |f^\sharp _{p_k}(z, u + i(|z|_\ell ^2 + o_{\text {wt}}(3)))|^2_\ell \nonumber \\  &   \quad = |\phi ^\sharp _{p_k}(z, u + i(|z|_\ell ^2 + o_{\text {wt}}(3)))|^2_{\ell ' - \ell } , \end{aligned}$$

where $u:={{\,\textrm{Re}\,}}w.$ Note that in the expression $u + i(|z|_\ell ^2 + o_{\text {wt}}(3)),$ both *u* and $|z|_\ell ^2$ are of weighted degree 2. For $m\in {{\mathbb {N}}},$ we collect terms of weighted degree *m* in the power series expansion of (2.20) and get:

$$\begin{aligned}  &   {{\,\textrm{Im}\,}}g^{\sharp (m)}_{p_k}(z, u + i|z|_\ell ^2) -2{{\,\textrm{Re}\,}}\big \langle f_{p_k}^{\sharp (m-1)}(z, u + i|z|_\ell ^2),\overline{z}\big \rangle _\ell \\  &   \quad = \Big (\big |\phi ^\sharp _{p_k}(z, u + i|z|_\ell ^2)\big |^2_{\ell ' - \ell }\Big )^{(m)}\! +\!H(g^{\sharp (h)}_{p_k}|_{h< m},\!f^{\sharp (h)}_{p_k}|_{h< m-1},\!(|\phi ^\sharp _{p_k}|_{\ell '-\ell }^2)^{(h)}|_{h< m} ), \end{aligned}$$

where *H* is a polynomial of weighted degree *m*,  consisting of elements from lower-degree terms $g^{\sharp (h)}_{p_k},$ $f^{\sharp (h)}_{p_k},$ and $(|\phi ^\sharp _{p_k}|_{\ell '-\ell }^2)^{(h)}.$ By induction, *H* is bounded. The term $o_{\text {wt}}(3)$ is only allowed to appear in these lower-degree terms and is therefore absorbed into *H*. Finally, the claim (2.18) can be established by using a method similar to that in the proof of [15, Lemma 6].

By passing to a subsequence, we assume that $(f_{p_k}^\sharp , g_{p_k}^\sharp )$ converges to formal maps $(f^\sharp ,g^\sharp )$ and $|\phi ^{\sharp }|^2_{\ell '-\ell }$ also converges to a formal function. (When $\ell '=\ell ,$ $(f^\sharp ,\phi ^{\sharp }, g^\sharp )$ is a formal map from $M_\ell$ into ${{\mathbb {H}}}^N_\ell .$ According to results from Meylan–Mir–Zaistev [19, 20], $F^\sharp =(\widetilde{f}^\sharp ,g^\sharp )$ is convergent.) Write ${\widetilde{f}}(z,w)$ and *g*(*z*, *w*) as series:

$$\begin{aligned} {\widetilde{f}}(z,w) = \sum _{\alpha +\beta>0} {\widetilde{f}}_{\alpha ,\beta }(z) w^\beta \quad \text {and} \quad g(z,w) = \sum _{\alpha +\beta >0} g_{\alpha ,\beta }(z) w^\beta , \end{aligned}$$

where $\widetilde{f}_{a,\beta }$ and $g_{\alpha ,\beta }$ are homogeneous holomorphic polynomials of degree $\alpha$ in *z*. We will prove the theorem by contradiction. Assume that $g \not \equiv 0.$ Then there exist $\alpha _0,\beta _0 \in {{\mathbb {N}}}$ such that $g_{\alpha _0,\beta _0}(z) \not \equiv 0$, and $g_{\mu \nu }(z) \equiv 0$ on ${\mathcal {S}}(\alpha _0,\beta _0):=\{(\mu ,\nu )\mid \mu + 2\nu< \alpha _0 + 2\beta _0, \text { or } \mu + 2\nu =\alpha _0 + 2\beta _0 \text { with } \nu < \beta _0\}.$ From (2.15), we have

2.21 $$\begin{aligned} g_{p_k}-\big (\lambda _k^{2}+ 2i\langle {\widetilde{f}}_{p_k},\overline{\textbf{a}}_k\rangle _E\big )g^\sharp _{p_k}=\lambda _k^{-2}(r_k-i|\textbf{a}_k|^2_E)g_{p_k}g^\sharp _{p_k}. \end{aligned}$$

Collecting holomorphic terms in (*z*, *w*) of bi-degree $(\alpha _0,\beta _0+1)$ in the power series expansion at 0 of (2.21), and let $w=1,$ we get that

2.22 $$\begin{aligned}  &   (g_{p_k})_{\alpha _0,\beta _0+1}(z)-\lambda _k^{2}(g^\sharp _{p_k})_{\alpha _0,\beta _0+1}(z)- 2i\big (\langle {\widetilde{f}}_{p_k},\overline{\textbf{a}}_k\rangle _E g^\sharp _{p_k}\big )_{\alpha _0,\beta _0+1}(z)\nonumber \\  &   \quad =\frac{(r_k-i|\textbf{a}_k|^2_E)}{\lambda _k^{2}}\Big ((g_{p_k})_{\alpha _0,\beta _0}(z)(g^\sharp _{p_k})_{0,1}(z)\nonumber \\  &   \quad +\sum _{\scriptscriptstyle (\mu ,\nu )\in {\mathcal {S}}(\alpha _0,\beta _0)}(g_{p_k})_{\mu \nu }(z)(g^ \sharp _{p_k})_{\alpha _0-\mu ,\beta _0+1-\nu }(z)\Big ). \end{aligned}$$

Notice that the left-hand side of (2.22) is uniformly bounded because of (2.14) and (2.18). It follows from $g_{\alpha _o,\beta _0}(z)\not \equiv 0$ that $(g_{p_k})_{\alpha _o,\beta _0}(z)\not \equiv 0$ for sufficiently large *k*. Moreover, $(g^\sharp _{p_k})_{0,1}(z)=1,$ $(g_{p_k})_{\mu \nu }(z)\rightarrow 0$ for all $\mu + 2\nu < \alpha _0 + 2\beta _0$ or $\mu + 2\nu =\alpha _0 + 2\beta _0$ with $\nu < \beta _0$ as $k\rightarrow \infty .$ Drawing from these observations, we obtain

$$\begin{aligned} |r_k-i|\textbf{a}_k|^2_E|\le C\lambda _k^2, \end{aligned}$$

for some constant *C* independent of *k*. By passing to a subsequence, we assume that

2.23 $$\begin{aligned} \frac{r_k-i|\textbf{a}_k|^2_E}{\lambda _k^2}\rightarrow C_0. \end{aligned}$$

Taking $k\rightarrow \infty$ in (2.21) yields in the formal sense that

2.24 $$\begin{aligned} g=(C_0g+2i\langle \widetilde{f},\overline{\textbf{a}}\rangle _E)g^\sharp . \end{aligned}$$

It is clear that $C_0g + 2i\langle {\widetilde{f}}, \overline{\textbf{a}}\rangle _E \not \equiv 0,$ otherwise, by (2.24), $g \equiv 0.$ Hence $g^\sharp$ is the quotient of two real analytic functions. By a simple application of the Artin approximation theorem, we deduce that $g^\sharp$ is convergent. Furthermore, from (2.15), we have

$$\begin{aligned} \lambda _k^2{\widetilde{f}}_{p_k}-\textbf{a}_kg_{p_k}=\big (\lambda _k^{2}+ 2i\langle {\widetilde{f}}_{p_k},\overline{\textbf{a}}_k\rangle _E +\lambda _k^{-2}(r_k-i|\textbf{a}_k|^2_E)g_{p_k}\big ){\widetilde{f}}^\sharp _{p_k}(\lambda _k\widetilde{A}_k), \end{aligned}$$

which implies

$$\begin{aligned} \big |\lambda _k^2{\widetilde{f}}_{p_k}-\textbf{a}_kg_{p_k}\big |_E^2&=\big |\lambda _k^{2}+ 2i\langle {\widetilde{f}}_{p_k},\overline{\textbf{a}}_k\rangle _E +\lambda _k^{-2}(r_k-i|\textbf{a}_k|^2_E)g_{p_k}\big |^2\big |{\widetilde{f}}^\sharp _{p_k}(\lambda _k\widetilde{A}_k)\big |_E^2\\&=\lambda _k^{2}\big |\lambda _k^{2}+ 2i\langle {\widetilde{f}}_{p_k},\overline{\textbf{a}}_k\rangle _E +\lambda _k^{-2}(r_k-i|\textbf{a}_k|^2_E)g_{p_k}\big |^2\big |{\widetilde{f}}^\sharp _{p_k}\big |_E^2. \end{aligned}$$

Hence we have

2.25 $$\begin{aligned} \frac{|\textbf{a}_k|_E^2}{\lambda _k^2}|g_{p_k}|^2= &   2{{\,\textrm{Re}\,}}\langle {\widetilde{f}}_{p_k},\overline{\textbf{a}}_k\,\overline{g}_{p_k}\rangle _E+\lambda _k^2|\widetilde{f}_{p_k}|_E^2\nonumber \\  &   +\big |\lambda _k^{2}+ 2i\langle {\widetilde{f}}_{p_k},\overline{\textbf{a}}_k\rangle _E +\lambda _k^{-2}(r_k-i|\textbf{a}_k|^2_E)g_{p_k}\big |^2\big |{\widetilde{f}}^\sharp _{p_k}\big |_E^2.\qquad \end{aligned}$$

From (2.18), the right-hand side of above is uniformly bounded. Since $g\not \equiv 0,$ it implies

$$\begin{aligned} \big ||\textbf{a}_k|_E^2\big |\le C\lambda _k^2 \end{aligned}$$

for some constant *C* independent of *k*. By passing to a subsequence, we assume that

$$\begin{aligned} |\textbf{a}_k|_E^2/\lambda _k^2\rightarrow C_1. \end{aligned}$$

Compare it with (2.23), we have $C_1=-{{\,\textrm{Im}\,}}C_0$ and $r_k/\lambda _k^2\rightarrow {{\,\textrm{Re}\,}}C_0.$ Letting $k\rightarrow \infty$ in (2.25), it follows from (2.14) that

$$\begin{aligned} C_1|g|^2=2{{\,\textrm{Re}\,}}\langle {\widetilde{f}},\overline{\textbf{a}}\,\overline{g}\rangle _E +\big |C_0g+2i\langle \widetilde{f},\overline{\textbf{a}}\rangle _E\big |^2\big |{\widetilde{f}}^\sharp \big |_E^2. \end{aligned}$$

Applying the Artin approximation theorem once more, we conclude that $\big |{\widetilde{f}}^\sharp \big |_E^2$ is convergent. Combining it with (2.24), we have

$$\begin{aligned} C_1|g|^2=|g|^2{{\,\textrm{Im}\,}}\Big (\frac{1}{g^\sharp }-C_0\Big ) +\frac{|g|^2}{|g^\sharp |^2}\big |{\widetilde{f}}^\sharp \big |_E^2, \end{aligned}$$

which gives

2.26 $$\begin{aligned} {{\,\textrm{Im}\,}}g^\sharp =\big |{\widetilde{f}}^\sharp \big |_E^2 \end{aligned}$$

on *U*. Observe that $\big |\widetilde{f}^\sharp _{p_k}\big |_E^2 = |z|_\ell ^2 + O(|(z,w)|^3).$ Differentiating both sides of (2.26) twice with respect to $\frac{\partial ^2}{\partial z_1 \partial \overline{z}_1}$ and evaluating at 0,  we obtain $0 = -1,$ which is a contradiction, thereby completing the proof. $\square$

We proceed the proof of Theorem 1.1 from the following theorem:

### Theorem 2.2

Assume that $\ell =\ell '.$ If *F* is transversally flat,  i.e.,  $g\equiv 0,$ then *F* is totally degenerate.

We now proceed to the proof of Theorem 2.2, which, in conjunction with Theorem 1.2, will allow us to complete the proof of Theorem 1.1 under the assumption that $M_\ell$ is real analytic.

### Proof of Theorem 2.2 and Theorem 1.1

For the sake of clarity, we denote $F=(\widetilde{f}, g):=(f_1, \ldots , f_{N-1}, g)$ throughout this proof. Then $g\equiv 0$ implies that

2.27 $$\begin{aligned} \sum _{j=1}^\ell |f_j|^2=\sum _{j=\ell +1}^{N-1}|f_j|^2 \quad \text {on } M_\ell . \end{aligned}$$

If ${\widetilde{f}}\equiv 0,$ then the proof is complete. If ${\widetilde{f}}\not \equiv 0,$ there exist a $1\le j\le \ell$ and an $\ell +1\le h\le N-1$ such that $f_j\not \equiv 0$ and $f_h\not \equiv 0.$ Without loss of generality, we can assume $f_1\not \equiv 0$ and $f_{N-1}\not \equiv 0.$ Let $S:=\{(z,w)\in {\mathbb {C}}^n\mid f_1+f_{N-1}=0\}$ and

$$\begin{aligned} {\widehat{F}}:=\left( \frac{f_2}{f_1+f_{N-1}},\ldots ,\frac{f_{N-2}}{f_1+f_{N-1}},\frac{i(f_1-f_{N-1})}{f_1+f_{N-1}}\right) . \end{aligned}$$

It is easy to verify that ${\widehat{F}}(M_\ell {\setminus } S)\subset {\mathbb {H}}_{\ell -1}^{N-2}.$ If $S\cap M_\ell \subsetneq M_\ell ,$ meaning $f_1+f_{N-1}\not \equiv 0$ on $M_\ell ,$ then $M_\ell \setminus S$ remains a real $(2n-1)$-dimensional hypersurface of signature $\ell .$ According to [2, Theorem 1.1], either ${\widehat{F}}({\widehat{V}})\subset {\mathbb {H}}_{\ell -1}^{N-2}$ for some ${\widehat{V}}\subset {\mathbb {C}}^n$ or ${\widehat{F}}$ is a locally embedding generically. The latter is impossible since the signature of ${\mathbb {H}}_{\ell -1}^{N-2}$ is one less than that of $M_\ell .$ The former implies that

$$\begin{aligned} {{\,\textrm{Im}\,}}\frac{i(f_1-f_{N-1})}{f_1+f_{N-1}}=-\sum _{j=2}^\ell \Big |\frac{f_j}{f_1+f_{N-1}}\Big |^2+\sum _{j=\ell +1}^{N-2}\Big |\frac{f_j}{f_1+f_{N-1}}\Big |^2 \quad \text {on } {\widehat{V}}\subset {\mathbb {C}}^n\setminus S, \end{aligned}$$

which yields

$$\begin{aligned} -\sum _{j=1}^\ell |f_j|^2+\sum _{j=\ell +1}^{N-1}|f_j|^2\equiv 0 \end{aligned}$$

on ${\widehat{V}}\subset {\mathbb {C}}^n\setminus S,$ and thus on $V\subset {\mathbb {C}}^n$ since $f_j$ are holomorphic functions. On the other hand, if $S\cap M_\ell = M_\ell ,$ i.e., $f_1+f_{N-1}\equiv 0$ on $M_\ell$ and hence on ${\mathbb {C}}^n$ from the uniqueness principle for holomorphic functions, then we obtain

$$\begin{aligned} \sum _{j=2}^\ell |f_j|^2=\sum _{j=\ell +1}^{N-2}|f_j|^2 \quad \text {on } M_\ell . \end{aligned}$$

Repeating the same process $\ell -2$ times if necessary as below of (2.27), we conclude that there exists $V\subset {\mathbb {C}}^n$ such that $F(V)\subset {\mathbb {H}}_\ell ^N.$

Now assume $M_\ell$ is just smooth and there is a holomorphic map *F* from a neighborhood *U* of $M_\ell$ in ${{\mathbb {C}}}^{n}$ into the hyperquadric ${{\mathbb {H}}}^N_\ell$ which is defined by $\widetilde{\rho }(\tilde{z},\tilde{w})={{\,\textrm{Im}\,}}\tilde{w}-|\tilde{w}|^2_\ell .$ When *F* is not totally degenerate, then $\widetilde{\rho }\circ F\not \equiv 0$ over *U*,  which defines a real analytic variety of codimension one containing $M_\ell .$ Since $M_\ell$ is smooth and also has real codimension one, by the Malgrange theorem [18, Proposition 3.11], $M_\ell$ is real analytic. This completes the proof of Theorem 1.1. $\square$

## Data Availability

There is no data associated to this work.

## References

[1] Baouendi, M.S., Ebenfelt, P., Huang, X.: Holomorphic mappings between hyperquadrics with small signature difference. Am. J. Math. **133**(6), 1633–1661 (2011)

[2] Baouendi, M.S., Ebenfelt, P., Rothschild, L.P.: Transversality of holomorphic mappings between real hypersurfaces in different dimensions. Commun. Anal. Geom. **15**(3), 589–611 (2007)

[3] Baouendi, M.S., Huang, X.: Super-rigidity for holomorphic mappings between hyperquadrics with positive signature. J. Differ. Geom. **69**(2), 379–398 (2005)

[4] Baouendi, M.S., Rothschild, L.P.: Geometric properties of mappings between hypersurfaces in complex space. J. Differ. Geom. **31**(2), 473–499 (1990)

[5] Chern, S.S., Moser, J.K.: Real hypersurfaces in complex manifolds. Acta Math. **133**, 219–271 (1974)

[6] Ebenfelt, P., Huang, X., Zaitsev, D.: The equivalence problem and rigidity for hypersurfaces embedded into hyperquadrics. Am. J. Math. **127**(1), 169–191 (2005)

[7] Ebenfelt, P., Rothschild, L.P.: Transversality of CR mappings. Am. J. Math. **128**(5), 1313–1343 (2006)

[8] Ebenfelt, P., Son, D.N.: CR transversality of holomorphic mappings between generic submanifolds in complex spaces. Proc. Am. Math. Soc. **140**(5), 1729–1738 (2012)

[9] Ebenfelt, P., Son, D.N.: Transversality of holomorphic mappings between real hypersurfaces in complex spaces of different dimensions. Ill. J. Math. 56(1):33–51 (2012)

[10] Fornaess, J.E.: Biholomorphic mappings between weakly pseudoconvex domains. Pac. J. Math. **74**(1), 63–65 (1978)

[11] Huang, X.: Schwarz reflection principle in complex spaces of dimension two. Commun. Partial Differ. Equ. **21**(11–12), 1781–1828 (1996)

[12] Huang, X.: On a linearity problem for proper holomorphic maps between balls in complex spaces of different dimensions. J. Differ. Geom. **51**(1), 13–33 (1999)

[13] Huang, X., Pan, Y.: Proper holomorphic mappings between real analytic domains in . Duke Math. J. **82**(2), 437–446 (1996)

[14] Huang, X., Zhang, Y.: On a CR transversality problem through the approach of the Chern–Moser theory. J. Geom. Anal. **23**(4), 1780–1793 (2013)

[15] Huang, X., Zhang, Y.: On the CR transversality of holomorphic maps into hyperquadrics. In: Complex Geometry and Dynamics. Abel Symposium, vol. 10, pp. 139–155. Springer, Cham (2015)

[16] Lamel, B., Mir, N.: Remarks on the rank properties of formal CR maps. Sci. China Ser. A **49**(11), 1477–1490 (2006)

[17] Lamel, B., Mir, N.: Convergence and divergence of formal CR mappings. Acta Math. **220**(2), 367–406 (2018)

[18] Malgrange, B.: Ideals of Differentiable Functions. Tata Institute of Fundamental Research Studies in Mathematics, vol. 3. Tata Institute of Fundamental Research, Bombay; Oxford University Press, London (1967)

[19] Mir, N.: Convergence of formal embeddings between real-analytic hypersurfaces in codimension one. J. Differ. Geom. **62**(1), 163–173 (2002)

[20] Meylan, F., Mir, N., Zaitsev, D.: Approximation and convergence of formal CR-mappings. Int. Math. Res. Not. **4**, 211–242 (2003)

